# Round the Hospitals

**Published:** 1918-11-23

**Authors:** 


### ROUND THE HOSPITALS.

Nowhere is the relaxation of "Dora's" grip upon the community more joyously celebrated than in the hospitals. Lofty windows, long remorselessly veiled or coloured, have been relieved of curtains, brown paper, stain, or other impediment to vision with the utmost celerity. Sunshine and moonshine stream once more down the long wards, and even the tiny children rejoice in the new aspect of affairs. So great is the enthusiasm that in many places all blinds are kept up through the evening just to emphasise the difference. The restrictions have pressed very hardly indeed on institutions facing the sea, especially those like the Royal Sea Bathing Infirmary at Margate, provided with open-

**ROUND THE HOSPITALS**—*(continued)*.

air wards looking out seawards. It is incredible how much can be done in the dark when the staff is once accustomed to it, but the extra troubles and trials caused by the lighting restrictions will never be forgotten by this generation of nurses.

THE presentation by Lady Simon of the medals and certificates gained by the probationers in their final examination at the Whipps Cross Infirmary and War Hospital, at Leyton, was an unusually stirring affair, from the fact that it took place in Armistice Week, when the hospital was *en fête*. The gold medal and certificate for the highest number of marks was gained by Nurse Maddock, the silver medal and certificate by Nurse Marsh, and the bronze medal and certificate by Nurse Clarke. Thirty-two nurses received certificates. The standard of training has been well maintained in the teeth of all difficulties, and the presence of the wounded has been felt as a great privilege.

THE "Peace Supper" on November 11 at the Prince of Wales's Hospital, Cardiff, is a sample of many such festivities held all over the kingdom, and was particularly lively and well carried out. The supper was given to all the patients in the hospital by Mrs. Cadogan, and was followed by a capital rendering of "The Mollusc." Badges were presented to eleven members of the voluntary staff who have put in twelve months' service or over, and a jewelled badge, with a bouquet from the staff, was presented to the Commandant, Miss Clara Deacon, by the senior V.A.D. nurse. Since the hospital was started 774 patients, of whom forty-four were officers, have been dealt with and fitted with limbs.

MEMBERS of the College of Nursing should find its Demobilisation Inquiry Bureau, which has just been set up, of great help in securing suitable appointments as and when they return from war service. The Council hope to be aided in their efforts in this direction by matrons, superintendents, and others keeping them advised of any vacancies upon their staff, and by nurses requiring posts communicating in good time with the secretary, giving their full name, if possible their registration number, and any other particulars likely to be of value. The Council are to be congratulated on their promptitude in establishing this Bureau, which was set up within two days of the declaration of the Armistice.

THE Bureau which is now in process of organisation is likely to be inundated with appeals for demobilised nursing sisters long before any are set free from their military duties. But it is of the utmost importance to get the machinery into working order, stimulate the demand, and educate public opinion on the subject of salaries, before the rush of home-coming nurses begins. To be quite outspoken we have our doubts whether gratitude towards the Nursing Service in time of war will restrain the public or the employers from trying to get nurses on the cheap in the dawn of peace. A proportion of military nurses will, it is true, retire from their labours when they are no longer required for the wounded. But the vast majority will be wanting fresh work. And from the outset the College will let it be understood that they are not to be exploited.

THE first Peace Investiture, held in the Ball-room of Buckingham Palace on November 16, was one of the most notable events in a very notable week. Everyone was in high spirits and very happy to be numbered amongst those specially honoured at this auspicious time. Custom should not make us oblivious of the grandeur of these solemn occasions. Never in the history of the world have there been gatherings more splendidly representative of British valour, more truly democratic than these when the King invests men and women, chosen out of all classes for conspicuous gallantry, with the insignia of the historic Orders into which they have been admitted in visible token of the nation's pride in their prowess.

THE KING bestowed the Royal Red Cross, First Class, on Matron Vida MacLean, New Zealand Army Nursing Service, and admitted the following to be Associates of the Order:—*Civil Nursing Service*: Sister Winifred Bimson, Sister Jessie Reid, and Sister Ethel Robinson. *New Zealand Army Nursing Service*: Sister Mary Christmas, Sister Jean Dodds, Sister Rose Fanning, Sister Matilda Fricker, Sister Emily Nutsey, Sister Alice Ingles, Sister Florence Siddells, Sister Mabel Wright, and Sister Carrie Young. *Voluntary Aid Detachment*: Mrs. Marjorie Monks.

THE KING has been pleased to award the Royal Red Cross to the following in recognition of their valuable services in connection with the war. The date of the award is June 3, 1918. FIRST CLASS.—Lady Superintendent Lallah Bessie Dunwoodie and Senior Nursing Sister Irene Annie McNally, of Queen Alexandra's Military Nursing Service for India; Matrons Alma Bennet, Annie Elizabeth Dowsley, and Teresa J. Dunne, of the Australian Army Nursing Service. SECOND CLASS.—Acting Senior Nursing Sister Winifred Mary Aldridge and Senior Nursing Sister Violet Isabel Lamb, of Queen Alexandra's Military Nursing Service for India; Senior Sisters Ethel B. Butler and Elizabeth Dalyell, and Acting Matron Elizabeth L. Horne, of the Australian Army Nursing Service.

THE Lambeth Guardians have fixed the salary of the new matron for whom they are advertising at £175 per annum, with board, lodging, washing, allowance for uniform, and £12 war bonus. The institution accommodates 956 patients. Readers will have noticed in our last issue the rate of salary fixed by the Cape Provincial Councils for matrons of public hospitals in the Dominion. Under this Ordnance matrons of second-grade hospitals (100 to 400 beds) receive £150 to £220 per annum, plus all allowances, and matrons of first-grade hospitals (over 400 beds) commence at £250 and advance to

ROUND THE HOSPITALS—(*continued*).

£350. The position of the Cape Town matron, even in the largest sized hospitals, responsible though it undoubtedly is, can hardly be compared in importance to that of a major London infirmary and training-school. How can the nursing profession in this country expect to secure the best women when one of the richest corporations in the richest city in the Empire has so low an opinion of what such women are worth?

The process of wearing-out matrons before their time is still going on all round us. The excuse that premature exhaustion is inevitable in war-time may be dismissed with contempt. The Lambeth Guardians have disguised the facts under a shower of compliments, but there can be no doubt that long prior to the war the strain on Miss Byles was too severe, and that had she been properly backed up by an efficient nursing committee, determined to relieve her, she would now be in the zenith of her working powers. During the war the position was aggravated in every possible way. It should have been met by drastic changes, undertaken in consultation with the matron herself. Instead of using their brains and money to ease the nursing administration, the Guardians followed the line of least resistance, and allowed their too efficient officer to be wrecked in the struggle. The small addition made to a very small pension can hardly be deemed adequate as an *amende honorable*.

A parting gift of £274 has been made to Miss Row on her resignation of office as lady superintendent of the East London Hospital for Children, Shadwell, held for twenty-three years. An even higher mark of the confidence and esteem in which she is held by her colleagues is afforded by the invitation accorded to her to join the board of the hospital on her retirement. It predicates knowledge of her possessing uncommon qualities, a spirit of open-mindedness to change, of reticence, and of generous consideration towards the matron who succeeds her in office, that this request should have been made. We venture to prophesy that the honorary work now undertaken by Miss Row will be second only in value to that achieved by her during the long period when she acted as mother to her institution.

A contemporary puts in a plea for recognition of the part played by religious nursing sisters in nursing the wounded and other works of mercy during the war. Like their lay *confrères*, they have stood bravely at their posts "through a terrific deluge of explosives," and have performed notable feats of endurance and devotion in the over-crowded and under-staffed hospitals in France. Catholic priests are urging the young women under their direction to embrace the profession of nursing. Pius X., it seems, was eminently in favour of women devoting themselves to the healing arts, though he was never reconciled to their speaking in public. There is certainly ample room for the "thousands of Catholic young women, filled with the spirit of sacrifice and animated by the purest love of God and their country," who are being invited to become nurses.

Miss Barton has done good service in setting tersely on record in the *Chelsea Infirmary Nurses' League Journal* a record of things achieved during the war, and we trust she will have many imitators. Every hospital, every Poor-Law infirmary, whose nurses have been privileged to take part in the great healing ministrations of this war ought to bring out particulars of what has been accomplished. Only so can the history of nursing in the war, regarded from this side, be adequately presented to the public. As principal matron of the 3rd London General Hospital, Miss Barton herself was one of the great organisers who led the way to victory. No fewer than ninety-two sisters and nurses, whose names were on the roll, were dispatched instantaneously on the order to mobilise. The Royal Patriotic School at Wandsworth was at once transformed into a modern military hospital with 500 beds, and has since quadrupled its accommodation.

Two of the Chelsea nurses were in the *Aquitania* when it was torpedoed—both happily saved after long exposure. Two were in charge of wards in the Royal Palace at Petrograd during the revolution. All "rose to the occasion," whether in long-drawn-out labours or in the vivid dangers of air raids. Very interesting is the record of those who, from ill-health or other reasons, have been compelled to abandon active war nursing. Sisters accustomed to command their wards have undertaken labourers' duties on farms. Others have become overseers at munition factories; some have taken up welfare work, and some have devoted themselves to preventive and health work among infants and children. The value of hospital training in fitting women for divers careers has never been more strikingly illustrated than during the last four years.

The examination of the Central Midwives Board for Scotland was held simultaneously at Edinburgh, Glasgow, and Dundee. There were twenty-three successful candidates in Edinburgh, thirty-seven in Glasgow, and eight in Dundee—sixty-eight in all. This number can hardly be said to meet the requirements of the Midwifery Service in the country in view of the fact that a good proportion of the candidates do not become practising midwives, and that the *bona-fide* midwives are destined, in the natural course of events, to gradual extinction.

The report of the June conference on maternity nursing in London reveals a state of things which will greatly astonish many people who thought they knew a good deal about midwifery among the poor. Briefly, it appears that the marked advance in the education and status of midwives during the last two decades has been unaccompanied by any improvement in the quality of the assistance available for the mothers after their confinement. Some midwives, but not all, give that maternity nursing for ten days prescribed by regulations. In

**ROUND THE HOSPITALS**—(*continued*).

the case of the women delivered by doctors and students the same ignorant and dirty service is still given which was all that could be had forty years ago. To this fact the experts ascribe a large proportion of that infantile mortality which disgraces the large towns.

The remedy can hardly lie in the direction of setting, as some would do, highly trained maternity nurses, who are to be debarred from holding the C.M.B. certificate, to follow on the steps of midwife, doctor, or student, and nurse the mother and infant for a fortnight, or even a month. It would be highly unreasonable to subordinate the trained nurse to the midwife, and, since it is estimated that the maternity nurse could take on but four cases at a time, it has to be recognised that there are not enough nurses to go round. But something might surely be done to bring into line and under control the women who so disastrously act as attendants after the birth. Elementary instruction of the kind which even the Indian *dai* can assimilate would remedy a great many evils. The younger women have all had some education, and could, we believe, be made to understand the dangers of dirt, the things they ought to do, and, more important, the things they ought not to do.

Well-deserved tribute to the work of the Leicester War Hospitals Committee was recently paid by Colonel L. K. Harrison, the C.O. of the 5th Northern General Hospital. He stated that the committee had been "the Father Christmas" in supplying the wounded in the hospitals with cigarettes, tobacco, newspapers, and games which had helped to make life in the hospitals endurable. The occasion was one which was intended to mark appreciation of the work of the Mayor and Mayoress of Leicester (Alderman and Mrs. North) on their retirement from office after a period of four years strenuous work. During this time the Mayor had been president of the War Hospitals Committee and had in countless ways been associated with the welfare of the military patients in the local hospitals. The officers, staff, and patients wished to present the Mayor with an illuminated address in appreciation of all that he had done for the hospitals during his Mayoralty. At the same time they desired the Mayoress to accept a silver cake-basket. She had organised a working party which had provided hundreds of thousands of articles for men at the Front and in hospitals. Mr. W. G. Gibbs (Hon. Sec. of the War Hospitals Committee) stated that the gift to the Mayoress might be accounted as coming entirely from the hospitals' staff and patients, and had been so inscribed.

At the recent annual meeting of the March Nursing Association, Cambridgeshire, the secretary, Mrs. Walton, made an interesting suggestion. She urged the meeting to consider the advisability of building as a war memorial a house to accommodate two nurses, with rooms which could be utilised as a school for mothers and an infant welfare centre. She suggested that the names of those who had lost their lives should be inscribed on stone tablets inserted in the walls. Such a memorial, designed to help the living for whose sake the dead had surrendered their lives, would, we feel sure, be eminently popular in country and town alike. The difficulty of obtaining nurses is aggravated in many districts by the poor accommodation available for them, and very few places can boast of a centre for advice, instruction, and aid.

